# Erlotinib: An enhancer of radiation therapy in nasopharyngeal carcinoma

**DOI:** 10.3892/etm.2013.1245

**Published:** 2013-08-02

**Authors:** HUAN-HUAN ZHANG, TAI-ZE YUAN, JIAN LI, YIN LIANG, LAI-JI HUANG, JIA-CAI YE, RONG-HUI ZHENG, GUO-FENG XIE, XIU-PING ZHANG

**Affiliations:** Department of Radiotherapy, Tumor Hospital of Guangzhou Medical College, Guangzhou, Guangdong 510095, P.R. China

**Keywords:** nasopharyngeal carcinoma, radiation, erlotinib

## Abstract

The aim of this study was to explore the effects of erlotinib combined with radiation on human nasopharyngeal carcinoma (NPC) radiosensitivity using the CNE1 and CNE2 cell lines. Human NPC cells were treated with erlotinib and/or radiation. The effect of erlotinib on the radiosensitivity of the cells was detected using a clonogenic cell survival assay. The rate of apoptosis and the cell cycle were evaluated using flow cytometry. An NPC xenograft model in NOD-SCID mice was used to evaluate the efficacy of the combination therapy of erlotinib with radiation. Erlotinib enhanced the sensitivity of the CNE1 and CNE2 cells to radiation, with sensitization enhancement ratios (SERs) of 1.076 and 1.109, respectively. Erlotinib combined with radiation induced G2/M phase cell cycle arrest in the two cell lines. The mouse tumor model demonstrated a significant reduction in NPC tumor volume in mice treated with erlotinib in combination with radiation when compared with that in mice treated with radiation alone. Erlotinib combined with radiation provoked G2-M phase cell cycle arrest, thereby enhancing the sensitivity of the NPC cells to radiation.

## Introduction

Nasopharyngeal carcinoma (NPC) is a relatively uncommon condition globally, with an incidence of less than 1 per 100,000 population (1). However, the disease occurs with much greater frequency in southern China, particularly in the province of Guangdong, where the incidence rises to 20–30 per 100,000 (1). Radiotherapy is the predominant treatment modality for this type of cancer. With the development of radiation technology and chemoradiotherapy, the 5-year overall and 5-year disease-free survival rates of patients with NPC have been reported to be 74.5 and 76.7%, respectively (2). Local-regional relapse and distant metastases remain the main causes of treatment failure in patients with NPC (2–4). These challenges make it necessary to explore new treatment modalities for NPC.

Radiotherapy is the radical treatment for patients with NPC. The use of chemotherapy drugs as radiotherapy sensitizers has been studied extensively in patients with NPC, including the use of fluorouracil (5-FU), cisplatin and taxanes (5,6). However, these drugs are limited in their clinical use due to severe acute toxicities, such as leukopenia and mucositis (5). In recent years, new molecular targeted therapies, including epidermal growth factor receptor (EGFR)-targeted therapy, have been widely recognized, and this recognition has been accompanied by significant breakthroughs in basic research and translational studies.

The EGFR is located primarily on cells of epithelial origin and is a transmembrane glycoprotein that belongs to the tyro-sine kinase factor family. The EGFR is overexpressed in the majority of human carcinomas, including breast, non-small cell lung, ovarian, bladder and head and neck cancer (7–10). Our previous study demonstrated that the EGFR was expressed in all patients with NPC, and it has been suggested that the over-expression of EGFR in NPC is correlated with an aggressive malignant progression and poor survival rates (11,12). These observations make NPC an appealing type of tumor in which to assay the effects of blocking the EGFR signaling pathway.

Tyrosine kinase inhibitors targeted against the EGFR, which block tyrosine kinase phosphorylation, have been shown to inhibit the EGFR-mediated proliferation of EGFR-rich cancer cells. Erlotinib is a small, reversible tyrosine kinase inhibitor that has been used in the treatment of several types of cancers. Erlotinib was designed to bind to the ATP pocket of the intracellular tyrosine kinase domain of the EGFR, inhibiting phosphorylation and thereby blocking the initiation of the intracellular cascade of transduction signals (13,14). Erlotinib has been shown to induce apoptosis and inhibit growth in several tumor cell lines *in vitro*, with the effects being associated with the induction of p27kip1 expression and blockade in the G1 phase of the cell cycle (13). In addition, erlotinib has been demonstrated to exert a substantial effect on the tumor growth of human HN5 xenografts in athymic mice and on pancreas-derived xenografts; the inhibitory effect was identified to be correlated with a reduction in the phosphorylation of extracellular-signal-regulated kinase (ERK), but not of Akt (14,15). *In vitro*, erlotinib has been shown to inhibit the proliferation of numerous types of cancer cells and enhance the antitumor effects of radiation (16).

The aim of this study was to investigate whether erlotinib is able to enhance the radiosensitivity of NPC and to explore its effects on tumor cell proliferation, apoptosis and the cell cycle in NPC cell lines.

## Materials and methods

### Cell culture and reagents

Human NPC cell lines (CNE1 and CNE2) were cultured in RPMI-1640 (Invitrogen Life Technologies, Carlsbad, CA, USA) supplemented with 10% fetal bovine serum (Hyclone, Logan, UT, USA) at 37°C in 5% CO_2_. Erlotinib was obtained from Roche (Basel, Switzerland). The apoptosis detection and cell cycle kits were purchased from Keygen Biotech Co., Ltd. (Nanjing, China). All other reagents were obtained from Sigma (St. Louis, MO, USA).

### Radiation technique

An X-radiometer was purchased from Rad Source Technologies, Inc. (Suwanee, GA, USA). Deep X-ray irradiation, with 160 kV voltage, 25 mA current, 0.3 mm copper filter and a dose rate of 623 cGy/min was performed. Six-well culture plates or 25 ml culture flasks were arranged in the center position of the apparatus.

### MTS assay

Exponentially growing NPC cells were seeded into 96-well plates at a density of 2,000 cells/well, incubated overnight at 37°C in 5% CO_2_ and treated with erlotinib at different concentrations for 72 h. Following the addition of 20 *μ*l of 5 mg/ml MTS to each well, the cells were incubated for 2 h at 37°C. The absorbance was read using a microplate reader (BioTek Instruments, Inc., Winooski, VT, USA) at a wavelength of 490 nm. Each experiment was performed in triplicate. The data were calculated as the mean values of three different experiments.

### Radiation cell survival assay

Exponentially growing NPC cells were plated in six-well plates, treated with 150 mmol erlotinib and incubated overnight at 37°C. The cells were then irradiated using X-rays at a dose rate of 623 cGy/min and were returned to the incubator for colony formation. After treating with erlotinib for 72 h, the cells were transferred to culture media without erlotinib. Following a period of 10–14 days, the clones were fixed in −20°C ethanol and stained with 1% crystal violet. Those clones that contained >50 cells were counted. Plating efficiency (PE) was calculated as the fraction of colonies counted divided by the number of cells plated without either erlotinib or ionizing radiation. The survival fraction (SF) was then calculated as the average number of colonies counted divided by the number of cells seeded multiplied by the PE. Using Sigmaplot™ 10.0 software (Systat Software, Inc., Chicago, IL, USA), the cell survival curves were fitted according to the survival data using single hit multi-target (SHMT) radiobiological models.

### Apoptosis and cell cycle analysis

NPC cells were treated with radiation, erlotinib (150 mmol/l) or the two in combination for different time periods. The cells were harvested and washed with ice-cold phosphate-buffered saline (PBS), fixed in 95% ethanol and stored at 4°C overnight. Following rehydration in PBS for 30 min at 4°C, cells were treated with 1% RNAase for 30 min at 37°C and stained with propidium iodide for 5 min. Cells were filtered through a nylon mesh with a pore size of 95 *μ*m and analyzed using a flow cytometer (Becton Dickinson, Franklin Lakes, NJ, USA).

### Animal experiments

Animal care and treatment was performed at the Animal Center of Guangzhou Medical College (Guangzhou, China). A total of 32 (16 males and 16 females) 6–7-week-old SCID mice were used in the study. Briefly, exponentially growing CNE2 cells (5×10^5^) were injected subcutaneously (s.c.) into the left hind flank of the mice on day 0. Eight days subsequent to the inoculation, the tumors reached a volume of 100–200 mm^3^. According to tumor volume, the animals were randomized into four groups, erlotinib (1.6 mg/day) alone, radiation (8 Gy) alone and erlotinib plus radiation. Erlotinib was administered by oral gavage once daily from day 8 to day 22. Radiation treatment was delivered once at a dose of 8 Gy using a custom lead block designed to expose only the tumor bed to radiation. Calipers were used to measure the length (L) and width (W) of the subcutaneous tumors. The tumor volume (TV) was calculated as: TV = (L×W^2^)/2. Mice were sacrificed one week subsequent to the end of the treatment and excised tumors were fixed in paraffin for immunohistochemical analysis. All animal studies were approved by the animal research ethics committee of Guangzhou Medical College (Guangzhou, China).

### Statistical analysis

SPSS version 12.0 statistical software (SPSS Inc., Chicago, IL, USA) was used for statistical analysis. The data were collected and calculated as the mean ± standard error (SE). Using one-way analysis of variance, the differences in the effect of each treatment alone and in combination were evaluated. P<0.05 was considered to indicate a statistically significant difference. Statistical significance was established by a post hoc least significant difference (LSD) pairwise comparison.

## Results

### Erlotinib inhibits cell proliferation of the NPC CNE2 cell line

**Figure 1.**
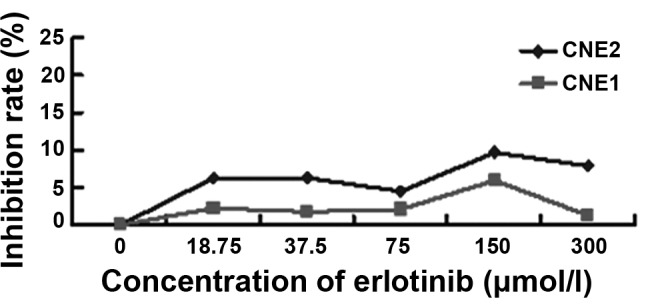
Inhibitory effect of erlotinib on nasopharyngeal carcinoma (NPC) cell lines. Cells were exposed for 72 h to increasing concentrations of erlotinib. Erlotinib inhibited proliferation of NPC CNE2 cells *in vitro,* as demonstrated by an MTS assay; however, this effect was not concentration-dependent. The data are presented as the mean ± standard error from three different experiments performed in triplicate.

The inhibition of NPC cell proliferation in the presence of erlotinib is shown in Fig. 1. The proliferation of the CNE2 cell line was inhibited by erlotinib but this was not concentration-dependent. However, the inhibition was not particularly effective in CNE2 cells, with a maximum inhibition rate of 9.74% at a concentration of 150 mmol. Similarly, the proliferation of the CNE1 cells was not inhibited by erlotinib.

### Erlotinib enhances radiosensitivity

**Figure 2.**
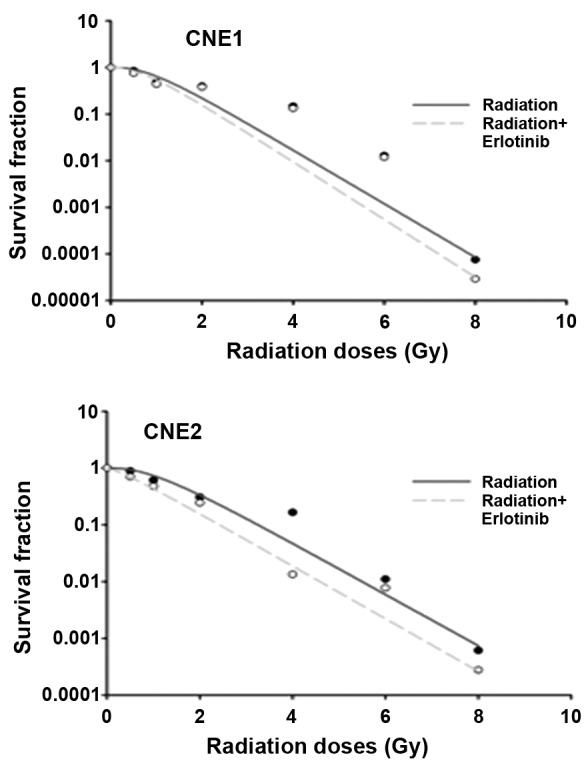
Cell survival curve fitted using the single-hit multi-target model in the nasopharyngeal carcinoma (NPC) cell line. CNE1 and CNE2 cells were exposed to increasing doses of radiation in the presence or absence of erlotinib. The figures show cell survival fitted using the click multiple target model. The data are presented as the mean ± standard error of the mean,.

To better understand the interaction of erlotinib and radiation in combination, a gold standard assessment of radiosensitivity was undertaken utilizing an *in vitro* colony formation assay. Fig. 2 depicts the radiation-survival curves for the two NPC cell lines, in which cells were exposed to 150 mmol erlotinib following radiation exposure at 0, 0.5, 1, 2, 4, 6 or 8 Gy. It was demonstrated that the survival fractions at 2 Gy (SF_2_) were 30.21 and 15.48% in the CNE2 cells treated with radiation alone and with the combination of erlotinib and radiation, respectively. Similarly, the data demonstrated a reduction in SF_2_ of 6.43% (from 21.90 to 15.47%) in the CNE1 cells following exposure to erlotinib and radiation. According to the single-hit multi-target model, this indicated that erlotinib enhanced the radiosensitivity of NPC cells (for the CNE1 and CNE2 cell lines), and the sensitization enhancement ratios (SERs) were 1.076 and 1.109, respectively.

### Erlotinib enhances radiation-induced apoptosis

**Figure 3.**
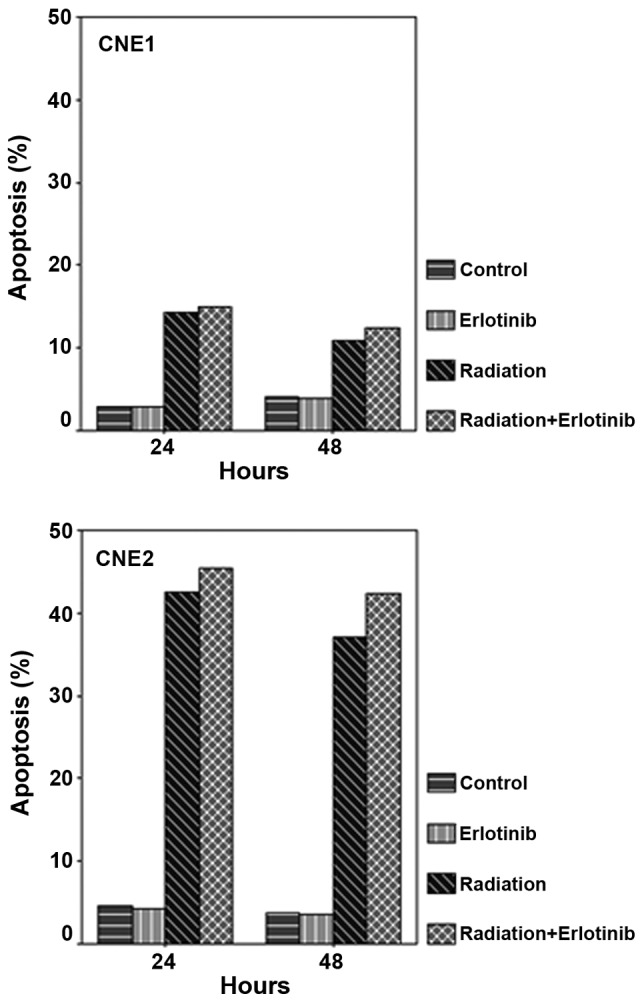
Effects of erlotinib combined with ionizing radiation on apoptosis in nasopharyngeal carcinoma cell lines. Histograms showing apoptosis in CNE1 and CNE2 cells that were treated with erlotinib (150 mmol/l) and/or ionizing radiation (Gy) for 24 or 48 h. The experiments were repeated three times and the values are the mean ± standard error of the mean.

In order to examine whether erlotinib induced an apoptotic response in NPC cells, NPC cells were exposed to erlotinib for 24 and 48 h in the presence or absence of radiation and flow cytometry using propidium iodide was performed to assess apoptosis. The results demonstrated that apoptosis was not induced in the CNE1 and CNE2 cells treated with erlotinib alone either for 24 or 48 h (Fig. 3). In addition, the effect of erlotinib on radiation-induced apoptosis was investigated. Statistically, the combined treatment of erlotinib with radiation significantly enhanced apoptosis in the CNE2 cells at 24 h (P=0.047). However, erlotinib combined with radiation did not enhance apoptosis in the CNE1 cells (P>0.05).

### Erlotinib induces G2/M cell cycle arrest

**Figure 4.**
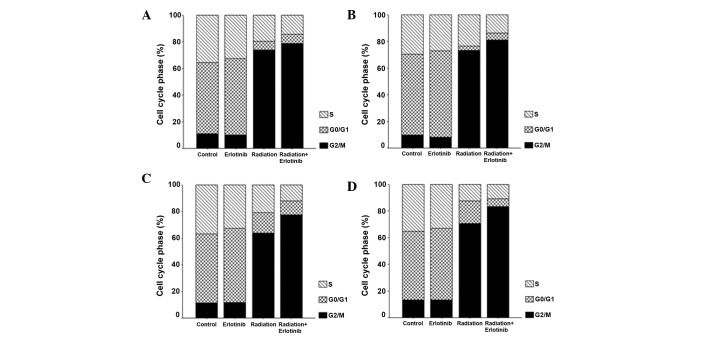
Effects of erlotinib and ionizing radiation on cell cycle arrest in nasopharyngeal carcinoma (NPC) cell lines. Histograms showing the effects of erlotinib (150 mmol/l) and/or ionizing radiation (Gy) in (A and B) CNE1 and (C and D) CNE2 cells at 24 (A and C) and 48 (B and D) h. The experiments were repeated three times and the results are presented as the mean ± standard error of the mean.

The capacity of erlotinib to inhibit cell cycle progression was evaluated using flow cytometric analyses (Fig. 4). Following exposure to erlotinib for 24 or 48 h, the accumulation of cells in the G2/M phase was not significantly different from the control in either the CNE1 or CNE2 cell lines. However, in the CNE2 cells treated with erlotinib for 48 h combined with radiation, the accumulation of cells in the G2/M phase (83.53%) was significantly higher than that of CNE2 cells treated with radiation alone (70.57%; P<0.05). Similarly, treatment with erlotinib combined with radiation in the CNE1 cells also led to a more marked G2/M phase arrest compared with treatment with radiation alone (P<0.05).

### Erlotinib augments the in vivo tumor response of NPC xenografts to radiation

**Figure 5.**
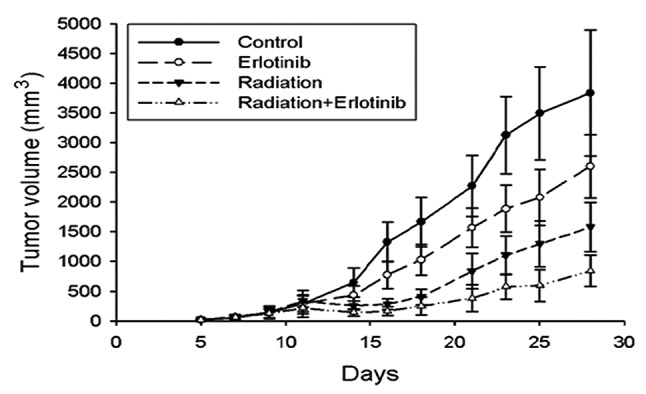
*In vivo* effect of erlotinib ± radiation on tumor volume in nasopharyngeal carcinoma (NPC) xenografts: Changes in tumor volume in NOD-SCID mice that were subcutaneously injected with 5×10^5^ CNE2 cells and treated with erlotinib (1.6 mg/day) alone, radiation (8 Gy) alone or erlotinib plus radiation. Control animals received vehicle alone. Data are presented as the mean ± standard error of the mean.

Human NPC (CNE2) cells were injected s.c. into athymic nude mice and allowed to grow for 8 days, prior to randomization of the mice into four groups. Eight days was the time interval required for the xenografts to reach 100∼200 mm^3^ in volume. As shown in Fig. 5, treatment with radiation alone or erlotinib alone produced a modest inhibition of tumor growth in the CNE2 xenografts. When combined with radiation, erlotinib enhanced the tumor growth inhibition profile over the 28-day observation period. Statistical analysis confirmed that the combination treatment resulted in a synergistic inhibitory effect on tumor growth in the CNE2 xenografts (P<0.05).

## Discussion

EGFR is a transmembrane tyrosine kinase growth factor receptor, whose molecular weight is 170 kD. It is divided into an extracellular amino terminal, a transmembrane segment and an intracellular carboxyl end. The intracellular region exhibits tyrosine kinase activity. A variety of tumors overexpress EGFR; in NPC tissue the expression rates have been shown to be 70.9–100% (11,17). High expression levels of EGFR in patients with NPC are correlated with a poor prognosis (17). Therefore, EGFR inhibitors may be of significance in the treatment of NPC. Erlotinib is an oral EGFR tyrosine kinase inhibitor and is currently one of the most extensively studied molecularly targeted agents. A clinical trial demonstrated that erlotinib enhanced the sensitivity to radiation therapy and improved survival rates in head and neck squamous cell carcinoma (18). A follow-up of this study performed in 2010 also demonstrated prolonged survival rates with minimal side effects (19). Several previous studies have demonstrated that erlotinib helps disrupt cell cycle pathways, as well as enhancing the sensitivity of cells to radiation (20). Tortora *et al* hypothesized that radiation therapy may enhance the effectiveness of erlotinib by creating a hypoxic environment at the tumor site (21).

The present study demonstrated that treatment of NPC cells with erlotinib alone had no significant effect on tumor cell proliferation. However, it was observed that erlotinib enhanced the radiosensitivity of the NPC cell lines. The CNE1 and CNE2 cells treated with erlotinib were shown to have SERs of 1.076 and 1.109, respectively, which were significantly higher than those of the cells treated with radiation therapy alone. One of the mechanisms by which erlotinib enhances the radio-sensitivity of NPC may be the induction of apoptosis of the tumor cells. Bai *et al* indicated that erlotinib induced apoptosis of A549 cells, a lung adenocarcinoma cell line, by regulating apoptosis-related genes (23). To confirm this hypothesis, we performed a cell cycle analysis of irradiated NPC cells that were exposed to erlotinib. It was observed that erlotinib alone was not able to induce apoptosis of tumor cells. However, the combination therapy of NPC cells with erlotinib and radiation led to CNE2 cell apoptosis (P=0.047). Based on *in vitro* studies in other types of cancer, we hypothesized that erlotinib enhanced radiation-induced cell cycle arrest in NPC cells (20). Earlier studies using lung cancer cell lines demonstrated that erlotinib induced cell cycle arrest at the G0/G1 phase (23,24). Erlotinib combined with radiotherapy induced cycle cell arrest at the G1 and G2/M phase, with a marked reduction in the S phase (24). However, it was observed in the present study that erlotinib alone had no significant effect on the cell cycle in NPC cells. Interestingly, erlotinib combined with ionizing radiation induced a significantly higher G2/M arrest in CNE1 and CNE2 cells compared with radiation alone.

An earlier study using H226 and UM-SCC6 tumor xenograft models demonstrated that erlotinib combined with RT dramatically inhibited tumor growth (24). Sarkaria *et al* showed that erlotinib and higher-dose radiation therapy resulted in an additive antitumor effect in a xenograft model of glioblastoma multiforme (25). In the present study a similar effect was observed in an NPC xenograft model using NOD-SCID mice. Erlotinib in combination with a single dose of irradiation led to a significant reduction in tumor volume compared with radiation alone.

In conclusion, the present study demonstrated that the EGFR tyrosine kinase inhibitor, erlotinib, combined with ionizing radiation induced cell cycle arrest at the G2/M phase and reduced tumor volume in a xenograft model. These results suggested that this may be a mechanism by which erlotinib enhances the sensitivity to radiation therapy in NPC. Further studies are required to elucidate other modes of action utilized by erlotinib.

## References

[1] Parkin DM, Whelan SL, Ferlay J, Raymond L, Young J (1997). Cancer Incidence in Five Continents. IARC Scientific Publications.

[2] Xiao WW, Huang SM, Han F (2011). Local control, survival, and late toxicities of locally advanced nasopharyngeal carcinoma treated by simultaneous modulated accelerated radiotherapy combined with cisplatin concurrent chemotherapy: long-term results of a phase 2 study. Cancer.

[3] Ng WT, Lee MC, Hung WM (2011). Clinical outcomes and patterns of failure after intensity-modulated radiotherapy for nasopharyngeal carcinoma. Int J Radiat Oncol Biol Phys.

[4] Song CH, Wu HG, Heo DS, Kim KH, Sung MW, Park CI (2008). Treatment outcomes for radiotherapy alone are comparable with neoadjuvant chemotherapy followed by radiotherapy in early-stage nasopharyngeal carcinoma. Laryngoscope.

[5] Lee AW, Lau WH, Tung SY, Chua DT, Chappell R (2005). Preliminary results of a randomized study on therapeutic gain by concurrent chemotherapy for regionally-advanced nasopharyngeal carcinoma: NPC-9901 Trial by the Hong Kong Nasopharyngeal Cancer Study Group. J Clin Oncol.

[6] Wee J, Tan EH, Tai BC (2005). Randomized trial of radiotherapy versus concurrent chemoradiotherapy followed by adjuvant chemotherapy in patients with American Joint Committee on Cancer/International Union against cancer stage III and IV nasopharyngeal cancer of the endemic variety. J Clin Oncol.

[7] Herbst RS, Langer CJ (2002). Epidermal growth factor receptors as a target for cancer treatment: the emerging role of IMC-C225 in the treatment of lung and head and neck cancers. Semin Oncol.

[8] Meche A, Cimpean AM, Raica M (2009). Immunohistochemical expression and significance of epidermal growth factor receptor (EGFR) in breast cancer. Rom J Morphol Embryol.

[9] Hirsch FR, Varella-Garcia M, Cappuzzo F (2009). Predictive value of EGFR and HER2 overexpression in advanced non-small-cell lung cancer. Oncogene.

[10] Leong JL, Loh KS, Putti TC, Goh BC, Tan LK (2004). Epidermal growth factor receptor in undifferentiated carcinoma of the nasopharynx. Laryngoscope.

[11] Yuan TZ, Li XX, Cao Y, Qian CN, Zeng MS, Guo X (2008). Correlation of epidermal growth factor receptor activation to metastasis-free survival of nasopharyngeal carcinoma patients. Ai Zheng.

[12] Yuan Y, Zhou X, Song J (2008). Expression and clinical significance of epidermal growth factor receptor and type 1 insulin-like growth factor receptor in nasopharyngeal carcinoma. Ann Otol Rhinol Laryngol.

[13] Moyer JD, Barbacci EG, Iwata KK (1997). Induction of apoptosis and cell cycle arrest by CP-358,774, an inhibitor of epidermal growth factor receptor tyrosine kinase. Cancer Res.

[14] Pollack VA, Savage DM, Baker DA (1999). Inhibition of epidermal growth factor receptor-associated tyrosine phosphorylation in human carcinomas with CP-358,774: dynamics of receptor inhibition in situ and antitumor effects in athymic mice. J Pharmacol Exp Ther.

[15] Ng SS, Tsao MS, Nicklee T, Hedley DW (2002). Effects of the epidermal growth factor receptor inhibitor OSI-774, Tarceva, on downstream signaling pathways and apoptosis in human pancreatic adenocarcinoma. Mol Cancer Ther.

[16] Chinnaiyan P, Huang S, Vallabhaneni G (2005). Mechanisms of enhanced radiation response following epidermal growth factor receptor signaling inhibition by erlotinib (Tarceva). Cancer Res.

[17] Ma BB, Poon TC, To KF (2003). Prognostic significance of tumor angiogenesis, Ki 67, p53 oncoprotein, epidermal growth factor receptor and HER2 receptor protein expression in undifferentiated nasopharyngeal carcinoma - a prospective study. Head Neck.

[18] Bonner JA, Harari PM, Giralt J (2006). Radiotherapy plus cetuximab for squamous-cell carcinoma of the head and neck. N Engl J Med.

[19] Bonner JA, Harari PM, Giralt J (2010). Radiotherapy plus cetuximab for locoregionally advanced head and neck cancer: 5-year survival data from a phase 3 randomized trial, and relation between cetuximab induced rash and survival. Lancet Oncol.

[20] Nyati MK, Morgan MA, Feng FY, Lawrence TS (2006). Integration of EGFR inhibitors with radiochemotherapy. Nat Rev Cancer.

[21] Tortora G, Gelardi T, Ciardiello F, Bianco R (2007). The rationale for the combination of selective EGFR inhibitors with cytotoxic drugs and radiotherapy. Int J Biol Markers.

[22] Bai XX, Mou XX, Jiang SJ (2010). Effects of Erlotinib on apoptosis in human pulmonary adenocarcinoma. Chinese Journal of Gerontology.

[23] Xiong X, Liu H, Fu L (2008). Antitumor activity of a new N-substituted thiourea derivative, an EGFR signaling-targeted inhibitor against a panel of human lung cancer cell lines. Chemotherapy.

[24] Huang S, Armstrong EA, Benavente S, Chinnaiyan P, Harari PM (2004). Dual-agent molecular targeting of the epidermal growth factor receptor (EGFR): combining anti-EGFR antibody with tyrosine kinase inhibitor. Cancer Res.

[25] Sarkaria JN, Carlson BL, Schroeder MA (2006). Use of an orthotopic xenograft model for assessing the effect of epidermal growth factor receptor amplification on glioblastoma radiation response. Clin Cancer Res.

